# Supplementary material for: A Likelihood Approach for Real-Time Calibration of Stochastic Compartmental Epidemic Models
Source: PLoS Comput Biol. 2017 Jan 17;13(1):e1005257. doi: 10.1371/journal.pcbi.1005257 (PMC5240920; doi:10.1371/journal.pcbi.1005257)
Supplement: S1 File — (TAR.GZ) [file pcbi.1005257.s014.tar.gz › HSPH_Online-SI-Revision/Additions/IPoi10/n100-extreme_noisy/n100-extreme_noisy_BENCH_table0.pdf]

1	R0	{2.76052, 16.3913}	{2.86118, 15.5722}	{{2.86118, 15.5722}, {2.86118, 15.5722}}	{3.64641, 4.99716}	inf	26 580.	24 313.9	{18 682., 28 050.}	12.0054	0.0716044	newly	10 934.9	10 734.	{8042., 12 499.}	13.3079	0.0675492	Reff	0.831027	1.05939	{0.539361, 1.6917}	48.4089	ar	92 090.	92 988.8	{92 812., 93 123.}	0.976002
2	R0	{2.18202, 15.1734}	{2.93978, 19.9817}	{{2.93978, 19.9817}, {2.93978, 19.9817}}	{34.7275, 31.689}	inf	17 999.	17 997.9	{10 052., 25 573.}	28.9148	0.132234	newly	8027.	5816.73	{3148., 9037.}	32.5915	0.191292	Reff	0.873157	0.48913	{0.277986, 0.944933}	54.5298	ar	84 264.	93 644.5	{93 473., 93 801.}	11.1322
3	R0	{2.57891, 12.5971}	{2.7943, 13.5962}	{{2.7943, 13.5962}, {2.7943, 13.5962}}	{8.35198, 7.93119}	inf	22 711.	19 770.8	{12 367., 26 994.}	21.5617	0.112837	newly	11 421.	9313.5	{5594., 13 379.}	25.8283	0.137708	Reff	0.727149	0.689771	{0.383266, 1.26548}	47.028	ar	90 302.	92 440.3	{92 260., 92 626.}	2.36797
4	R0	{2.5856, 9.28508}	{2.91001, 10.5991}	{{2.91001, 10.5991}, {2.91001, 10.5991}}	{12.5468, 14.152}	inf	23 144.	25 045.5	{20 505., 28 872.}	17.852	0.0947596	newly	15 345.	15 653.6	{11 730., 18 627.}	15.5964	0.081739	Reff	0.760942	1.00022	{0.523249, 1.78398}	50.1194	ar	90 312.	93 414.2	{93 238., 93 588.}	3.43504
5	R0	{2.4406, 12.1341}	{2.7943, 13.5962}	{{2.7943, 13.5962}, {2.7943, 13.5962}}	{14.4923, 12.0495}	inf	22 895.	23 078.8	{17 773., 27 180.}	14.0602	0.0660794	newly	12 782.	11 170.6	{7887., 13 684.}	15.9317	0.0818173	Reff	0.972189	0.835519	{0.460696, 1.45287}	38.8414	ar	88 597.	92 424.7	{92 275., 92 557.}	4.32035
6	R0	{2.72155, 15.4094}	{2.86118, 15.5722}	{{2.86118, 15.5722}, {2.86118, 15.5722}}	{5.13053, 1.0565}	inf	26 147.	19 588.6	{12 019., 26 990.}	26.6448	0.148819	newly	11 271.	7978.31	{4878., 11 482.}	30.9302	0.178133	Reff	0.880857	0.559761	{0.320395, 0.873461}	47.4722	ar	91 682.	92 999.2	{92 829., 93 130.}	1.43675
7	R0	{2.63781, 14.3935}	{2.85582, 17.2484}	{{2.85582, 17.2484}, {2.85582, 17.2484}}	{8.26481, 19.8346}	inf	24 939.	23 858.6	{17 478., 27 910.}	13.6924	0.0693851	newly	11 237.	9413.37	{7123., 11 235.}	16.6174	0.087709	Reff	0.790499	0.946747	{0.472524, 1.6981}	45.8542	ar	91 192.	92 980.7	{92 827., 93 121.}	1.96143
8	R0	{2.82391, 16.104}	{2.86118, 15.5722}	{{2.86118, 15.5722}, {2.86118, 15.5722}}	{1.3198, 3.30229}	inf	27 368.	22 336.7	{16 256., 28 007.}	19.1965	0.110906	newly	11 207.	9387.68	{6289., 12 577.}	22.204	0.123107	Reff	0.861801	0.733404	{0.379478, 1.28364}	42.5946	ar	92 664.	92 988.4	{92 849., 93 138.}	0.350115
9	R0	{2.80645, 14.6457}	{2.8939, 16.8013}	{{2.8939, 16.8013}, {2.8939, 16.8013}}	{3.11604, 14.7183}	inf	27 606.	24 834.	{16 028., 28 752.}	13.1727	0.0875202	newly	12 694.	10 574.7	{8362., 11 906.}	16.6956	0.0982593	Reff	0.94945	1.30278	{0.725877, 2.11107}	45.9906	ar	92 567.	93 259.9	{93 135., 93 407.}	0.748502
10	R0	{1.86213, 5.4271}	{2.83858, 10.6973}	{{2.83858, 10.6973}, {2.83858, 10.6973}}	{52.4373, 97.109}	inf	10 896.	23 305.5	{16 210., 27 935.}	114.944	0.324593	newly	11 765.	13 650.8	{8788., 17 255.}	27.2689	0.108988	Reff	0.763157	0.724734	{0.397543, 1.15965}	33.1767	ar	75 362.	92 816.8	{92 656., 92 973.}	23.1612
11	R0	{2.27349, 14.6338}	{2.8833, 17.1847}	{{2.8833, 17.1847}, {2.8833, 17.1847}}	{26.8226, 17.4316}	inf	19 721.	21 249.7	{14 581., 27 220.}	23.2428	0.0967939	newly	8893.	8044.89	{5240., 11 008.}	23.3284	0.115296	Reff	0.854264	0.629838	{0.349687, 1.32779}	44.2153	ar	85 805.	93 175.7	{93 039., 93 321.}	8.59004
12	R0	{2.77261, 14.1894}	{2.8833, 17.1847}	{{2.8833, 17.1847}, {2.8833, 17.1847}}	{3.99227, 21.1094}	inf	27 138.	24 524.4	{17 467., 28 734.}	12.7897	0.0801757	newly	12 745.	10 029.5	{8056., 11 594.}	21.3068	0.120073	Reff	0.865526	1.18306	{0.622908, 2.01894}	52.2305	ar	92 508.	93 195.1	{93 019., 93 362.}	0.74279
13	R0	{2.65402, 19.3935}	{2.81891, 18.2812}	{{2.81891, 18.2812}, {2.81891, 18.2812}}	{6.21284, 5.73543}	inf	25 765.	19 753.5	{12 871., 27 179.}	25.1956	0.13797	newly	8948.	7073.81	{4645., 10 234.}	26.2806	0.140926	Reff	0.987375	0.651852	{0.350616, 1.14606}	50.772	ar	91 184.	92 654.7	{92 516., 92 807.}	1.61286
14	R0	{1.75297, 4.45354}	{2.7943, 13.5962}	{{2.7943, 13.5962}, {2.7943, 13.5962}}	{59.4038, 205.29}	inf	10 727.	24 295.3	{20 303., 27 341.}	126.549	0.350017	newly	15 310.	12 203.4	{9828., 13 973.}	20.2914	0.103139	Reff	0.926059	1.00454	{0.561095, 1.56414}	38.1947	ar	71 263.	92 475.	{92 290., 92 643.}	29.7658
15	R0	{1.99928, 7.1501}	{2.96307, 14.3219}	{{2.96307, 14.3219}, {2.96307, 14.3219}}	{48.2069, 100.303}	inf	13 996.	26 223.1	{21 977., 29 551.}	89.8561	0.282561	newly	12 056.	12 584.7	{9709., 14 302.}	14.4047	0.069011	Reff	0.80563	1.06607	{0.544642, 1.67458}	48.7137	ar	79 602.	93 762.4	{93 594., 93 899.}	17.789
16	R0	{2.67398, 10.3134}	{2.96307, 14.3219}	{{2.96307, 14.3219}, {2.96307, 14.3219}}	{10.8112, 38.8669}	inf	23 578.	23 863.2	{18 048., 28 720.}	14.3846	0.0635046	newly	14 011.	10 673.	{7912., 13 508.}	23.8315	0.127165	Reff	0.705449	0.67648	{0.403926, 1.1358}	36.7183	ar	91 289.	93 795.5	{93 655., 93 919.}	2.74564
17	R0	{2.44634, 16.3226}	{2.97157, 19.3547}	{{2.97157, 19.3547}, {2.97157, 19.3547}}	{21.47, 18.5761}	inf	22 666.	25 513.3	{19 999., 29 581.}	20.8994	0.103023	newly	9419.	8914.	{6631., 10 532.}	14.4096	0.0837377	Reff	0.907103	0.905916	{0.474946, 1.47048}	39.8431	ar	88 710.	93 847.1	{93 705., 93 985.}	5.79084
18	R0	{2.02168, 10.1676}	{2.97157, 19.3547}	{{2.97157, 19.3547}, {2.97157, 19.3547}}	{46.9852, 90.3566}	inf	15 535.	24 829.5	{12 235., 29 457.}	69.8717	0.241953	newly	10 392.	9059.59	{5659., 10 623.}	13.8	0.0822406	Reff	0.996001	1.25533	{0.600941, 2.39247}	50.1114	ar	80 299.	93 860.2	{93 719., 93 977.}	16.8884
19	R0	{2.89969, 15.009}	{2.86118, 15.5722}	{{2.86118, 15.5722}, {2.86118, 15.5722}}	{1.32807, 3.75242}	inf	28 996.	24 760.9	{19 873., 28 082.}	14.6056	0.0777535	newly	12 862.	10 842.7	{8414., 12 584.}	15.7	0.0804099	Reff	0.881738	0.998727	{0.518417, 1.84065}	40.4914	ar	93 349.	93 010.3	{92 841., 93 150.}	0.362808
20	R0	{2.68933, 7.34928}	{2.83858, 10.6973}	{{2.83858, 10.6973}, {2.83858, 10.6973}}	{5.54971, 45.5558}	inf	24 920.	22 100.3	{10 119., 27 849.}	20.2711	0.13316	newly	20 039.	14 834.7	{10 024., 17 921.}	25.9708	0.15367	Reff	0.765733	1.36349	{0.621308, 2.37274}	84.1534	ar	91 574.	92 799.5	{92 684., 92 936.}	1.33828
21	R0	{2.15352, 9.28074}	{2.7943, 13.5962}	{{2.7943, 13.5962}, {2.7943, 13.5962}}	{29.755, 46.4991}	inf	17 876.	23 577.2	{15 769., 27 568.}	42.684	0.170252	newly	12 361.	12 146.7	{8990., 13 961.}	13.6977	0.0708786	Reff	0.8829	1.19769	{0.656772, 2.04842}	49.3868	ar	83 845.	92 442.2	{92 245., 92 624.}	10.2537
22	R0	{2.21953, 9.04387}	{2.9884, 12.8326}	{{2.9884, 12.8326}, {2.9884, 12.8326}}	{34.6411, 41.8928}	inf	18 399.	25 439.9	{20 444., 29 830.}	43.5384	0.167722	newly	13 126.	12 969.5	{10 021., 16 037.}	15.7934	0.0789153	Reff	0.851523	0.868152	{0.459257, 1.50974}	42.5275	ar	84 856.	93 971.5	{93 813., 94 139.}	10.7424
23	R0	{2.21987, 14.5347}	{2.93978, 19.9817}	{{2.93978, 19.9817}, {2.93978, 19.9817}}	{32.4303, 37.4758}	inf	18 816.	23 058.	{17 531., 28 818.}	28.122	0.109301	newly	8717.	7622.74	{5482., 10 073.}	19.8722	0.102882	Reff	0.868968	0.696366	{0.385758, 1.1917}	42.7634	ar	85 067.	93 605.6	{93 449., 93 741.}	10.0375
24	R0	{2.84268, 17.9659}	{2.76795, 19.6041}	{{2.76795, 19.6041}, {2.76795, 19.6041}}	{2.62886, 9.11839}	inf	27 580.	22 954.9	{17 265., 26 639.}	16.7696	0.090938	newly	10 377.	7832.92	{6036., 9397.}	24.5166	0.133134	Reff	0.920602	0.799873	{0.467645, 1.28892}	36.5806	ar	92 836.	92 196.	{92 006., 92 395.}	0.68937
25	R0	{2.52706, 10.3679}	{2.8833, 17.1847}	{{2.8833, 17.1847}, {2.8833, 17.1847}}	{14.097, 65.7491}	inf	22 248.	24 793.7	{15 967., 28 556.}	24.7638	0.118881	newly	13 481.	10 070.4	{7836., 11 531.}	25.2992	0.142617	Reff	0.761075	1.17904	{0.661198, 2.10913}	60.7115	ar	89 623.	93 142.2	{92 957., 93 310.}	3.9267
26	R0	{2.62835, 19.1088}	{2.97157, 19.3547}	{{2.97157, 19.3547}, {2.97157, 19.3547}}	{13.0584, 1.28684}	inf	24 689.	23 758.3	{17 257., 29 438.}	15.625	0.0756659	newly	8698.	8086.88	{6003., 10 436.}	18.2271	0.0887471	Reff	0.928833	0.706463	{0.398369, 1.18967}	43.716	ar	90 908.	93 835.7	{93 694., 93 976.}	3.22046
27	R0	{2.76831, 19.4155}	{2.8939, 16.8013}	{{2.8939, 16.8013}, {2.8939, 16.8013}}	{4.5367, 13.4645}	inf	25 708.	17 811.5	{11 036., 24 801.}	32.4571	0.187946	newly	8776.	6698.79	{4032., 9321.}	30.4078	0.169495	Reff	0.75287	0.4766	{0.296335, 0.624272}	48.3015	ar	92 175.	93 285.8	{93 138., 93 423.}	1.20513
28	R0	{2.96609, 16.8903}	{2.99939, 13.7076}	{{2.99939, 13.7076}, {2.99939, 13.7076}}	{1.12269, 18.8434}	inf	28 896.	20 403.4	{14 140., 28 167.}	29.796	0.170704	newly	11 093.	9235.67	{6141., 13 079.}	24.9196	0.129941	Reff	0.743925	0.549894	{0.319285, 0.790849}	48.4456	ar	94 024.	94 045.3	{93 899., 94 232.}	0.0984351
29	R0	{2.17046, 14.39}	{2.86095, 19.9929}	{{2.86095, 19.9929}, {2.86095, 19.9929}}	{31.8131, 38.9361}	inf	18 683.	25 136.	{21 487., 28 247.}	39.9645	0.152113	newly	8778.	8686.5	{6860., 9828.}	11.3677	0.0559365	Reff	0.946993	1.04227	{0.556741, 1.62476}	37.8419	ar	84 001.	93 006.8	{92 845., 93 172.}	10.7211
30	R0	{2.88772, 12.4647}	{2.91001, 10.5991}	{{2.91001, 10.5991}, {2.91001, 10.5991}}	{0.771889, 14.9671}	inf	28 167.	21 052.2	{14 035., 28 308.}	25.7435	0.157877	newly	14 527.	12 144.9	{7416., 17 895.}	25.9668	0.142744	Reff	0.836804	0.658893	{0.345535, 1.16831}	47.8907	ar	93 273.	93 384.4	{93 221., 93 552.}	0.148757
31	R0	{2.46831, 8.14308}	{2.9194, 10.1535}	{{2.9194, 10.1535}, {2.9194, 10.1535}}	{18.2753, 24.6887}	inf	22 336.	23 526.9	{10 130., 29 002.}	30.0384	0.156191	newly	17 282.	16 384.7	{11 099., 19 485.}	13.7887	0.0761026	Reff	0.883087	1.34006	{0.694496, 2.44818}	62.0032	ar	88 762.	93 437.1	{93 267., 93 600.}	5.26706
32	R0	{1.93236, 7.38987}	{2.9884, 12.8326}	{{2.9884, 12.8326}, {2.9884, 12.8326}}	{54.6503, 73.6512}	inf	13 853.	25 503.3	{20 760., 29 861.}	85.033	0.263037	newly	12 032.	12 805.	{9695., 15 909.}	17.0004	0.0702473	Reff	0.884615	0.768119	{0.460662, 1.104}	37.6905	ar	77 654.	93 991.7	{93 842., 94 138.}	21.039
33	R0	{2.82251, 17.6954}	{2.99911, 15.6207}	{{2.99911, 15.6207}, {2.99911, 15.6207}}	{6.25684, 11.7245}	inf	27 972.	24 847.4	{18 541., 29 952.}	14.8529	0.0771423	newly	10 722.	10 498.8	{7390., 13 131.}	17.744	0.0818547	Reff	1.00304	0.80686	{0.40566, 1.31967}	42.8466	ar	92 745.	94 046.7	{93 912., 94 199.}	1.40356
34	R0	{1.87083, 12.3994}	{2.76795, 19.6041}	{{2.76795, 19.6041}, {2.76795, 19.6041}}	{47.953, 58.1052}	inf	12 842.	23 803.6	{18 920., 26 968.}	85.3575	0.264119	newly	7135.	8270.18	{6431., 9522.}	20.1255	0.0794293	Reff	1.01449	0.889046	{0.477471, 1.40717}	34.0141	ar	76 282.	92 226.8	{92 031., 92 418.}	20.9025
35	R0	{2.95779, 16.3003}	{2.7346, 14.003}	{{2.7346, 14.003}, {2.7346, 14.003}}	{7.54584, 14.0936}	inf	29 124.	21 615.	{15 473., 26 420.}	25.7829	0.159004	newly	12 158.	10 165.9	{6637., 12 938.}	19.2117	0.116806	Reff	1.01659	0.839203	{0.420335, 1.47832}	41.084	ar	93 873.	91 879.	{91 701., 92 047.}	2.12418
36	R0	{2.18718, 14.261}	{2.8939, 16.8013}	{{2.8939, 16.8013}, {2.8939, 16.8013}}	{32.3119, 17.8129}	inf	18 672.	16 414.9	{9562., 25 875.}	27.6144	0.13876	newly	8858.	6134.86	{3431., 9753.}	36.1387	0.211147	Reff	0.915641	0.441857	{0.286004, 0.665018}	57.0086	ar	84 379.	93 242.2	{93 080., 93 420.}	10.504
37	R0	{2.44912, 9.31118}	{2.80387, 10.6536}	{{2.80387, 10.6536}, {2.80387, 10.6536}}	{14.4848, 14.4173}	inf	21 427.	22 916.9	{17 850., 27 448.}	15.6205	0.0661196	newly	14 344.	14 060.1	{10 763., 17 776.}	15.7237	0.0722471	Reff	0.796601	0.870396	{0.471919, 1.72884}	45.0046	ar	88 442.	92 547.	{92 364., 92 706.}	4.64151
38	R0	{2.05949, 5.72514}	{2.9884, 12.8326}	{{2.9884, 12.8326}, {2.9884, 12.8326}}	{45.1039, 124.145}	inf	16 011.	24 686.5	{14 122., 29 836.}	68.2872	0.275267	newly	16 782.	13 623.4	{10 579., 16 075.}	18.8215	0.136422	Reff	0.827297	1.33144	{0.659689, 2.30176}	69.963	ar	81 587.	93 964.3	{93 797., 94 104.}	15.1707
39	R0	{1.75505, 8.52404}	{2.96978, 18.8857}	{{2.96978, 18.8857}, {2.96978, 18.8857}}	{69.2134, 121.558}	inf	10 952.	24 665.7	{15 303., 29 624.}	132.445	0.394771	newly	8590.	8999.5	{6399., 10 933.}	20.6651	0.129371	Reff	0.957748	1.1081	{0.512168, 2.23396}	48.3779	ar	70 925.	93 832.9	{93 689., 93 974.}	32.2988
40	R0	{2.24724, 13.7861}	{2.86118, 15.5722}	{{2.86118, 15.5722}, {2.86118, 15.5722}}	{27.3197, 12.9558}	inf	18 516.	20 923.7	{12 857., 26 659.}	26.261	0.107965	newly	8935.	8650.45	{5467., 11 670.}	19.9827	0.0952847	Reff	0.839861	0.661493	{0.350208, 1.07689}	46.352	ar	85 257.	93 030.4	{92 861., 93 165.}	9.11765
41	R0	{1.77635, 8.62406}	{2.86118, 15.5722}	{{2.86118, 15.5722}, {2.86118, 15.5722}}	{61.0707, 80.5669}	inf	11 281.	24 254.	{19 249., 28 125.}	121.872	0.35581	newly	8585.	10 372.3	{7949., 12 369.}	29.5239	0.125161	Reff	0.87725	0.922389	{0.509347, 1.50644}	38.1717	ar	72 941.	92 988.9	{92 844., 93 174.}	27.4851
42	R0	{1.80397, 5.4932}	{2.80387, 10.6536}	{{2.80387, 10.6536}, {2.80387, 10.6536}}	{55.4278, 93.9416}	inf	10 692.	19 145.8	{12 623., 26 694.}	81.3827	0.247082	newly	11 817.	11 008.2	{6689., 17 411.}	28.144	0.133572	Reff	0.8332	0.603222	{0.349811, 1.06216}	46.5383	ar	73 232.	92 513.	{92 366., 92 701.}	26.3286
43	R0	{2.83096, 16.7077}	{2.93978, 19.9817}	{{2.93978, 19.9817}, {2.93978, 19.9817}}	{3.84393, 19.5958}	inf	27 789.	24 801.2	{17 868., 29 198.}	13.511	0.085543	newly	11 209.	8759.54	{7069., 10 201.}	21.8526	0.125307	Reff	0.932207	1.19919	{0.557294, 2.06146}	50.7158	ar	92 740.	93 637.7	{93 456., 93 812.}	0.96795
44	R0	{2.73678, 11.6021}	{2.9884, 12.8326}	{{2.9884, 12.8326}, {2.9884, 12.8326}}	{9.19402, 10.6058}	inf	23 763.	22 031.8	{14 502., 29 266.}	19.0567	0.0918864	newly	12 451.	10 619.6	{6573., 14 875.}	23.6109	0.121946	Reff	0.638655	0.558463	{0.320058, 0.856535}	36.2899	ar	92 003.	93 963.1	{93 815., 94 105.}	2.13052
45	R0	{2.14235, 6.98601}	{2.96307, 14.3219}	{{2.96307, 14.3219}, {2.96307, 14.3219}}	{38.3093, 105.008}	inf	15 339.	25 977.9	{22 263., 29 418.}	72.1601	0.241233	newly	13 010.	12 392.2	{10 265., 14 313.}	11.5756	0.0599941	Reff	0.718266	1.03608	{0.536019, 1.83115}	57.8738	ar	82 974.	93 771.7	{93 606., 93 896.}	13.0133
46	R0	{2.23409, 9.21289}	{2.7943, 13.5962}	{{2.7943, 13.5962}, {2.7943, 13.5962}}	{25.0755, 47.578}	inf	19 256.	23 422.3	{14 893., 27 154.}	33.4073	0.141236	newly	14 108.	12 028.8	{9116., 13 948.}	14.7713	0.0814443	Reff	0.983357	1.19327	{0.660517, 2.08332}	43.1577	ar	85 163.	92 432.9	{92 248., 92 623.}	8.53647
47	R0	{2.12258, 8.15265}	{2.91946, 11.8546}	{{2.91946, 11.8546}, {2.91946, 11.8546}}	{37.543, 45.4079}	inf	17 054.	25 519.6	{21 025., 28 864.}	54.3775	0.19382	newly	13 477.	14 409.4	{11 031., 16 819.}	16.664	0.0733059	Reff	0.871829	0.992456	{0.537122, 1.61052}	40.8883	ar	82 690.	93 466.6	{93 274., 93 620.}	13.0325
48	R0	{2.71771, 10.4636}	{2.9884, 12.8326}	{{2.9884, 12.8326}, {2.9884, 12.8326}}	{9.96022, 22.6404}	inf	26 340.	26 823.5	{21 510., 29 893.}	11.9029	0.0551628	newly	16 548.	14 516.2	{12 423., 16 109.}	12.278	0.0596153	Reff	0.927228	1.14089	{0.635722, 1.88039}	42.1197	ar	91 716.	93 976.4	{93 853., 94 092.}	2.46457
49	R0	{2.44369, 6.66477}	{2.98388, 9.60029}	{{2.98388, 9.60029}, {2.98388, 9.60029}}	{22.1055, 44.0453}	inf	18 124.	24 897.5	{18 727., 29 719.}	42.1273	0.155014	newly	14 957.	16 537.6	{11 766., 21 269.}	21.7523	0.0887392	Reff	0.592717	0.869859	{0.421563, 1.54992}	62.3629	ar	88 827.	93 948.9	{93 824., 94 094.}	5.76615
50	R0	{2.77383, 17.9775}	{2.97157, 19.3547}	{{2.97157, 19.3547}, {2.97157, 19.3547}}	{7.12877, 7.66069}	inf	25 584.	25 748.6	{20 605., 29 302.}	12.3049	0.066387	newly	9271.	9042.14	{6808., 10 649.}	12.5837	0.0667084	Reff	0.747852	0.926486	{0.474946, 1.62488}	46.7264	ar	92 334.	93 840.4	{93 688., 93 982.}	1.6315
